# Immigrant and refugee experiences in the Utah WIC program: a qualitative study

**DOI:** 10.1186/s12939-026-02765-7

**Published:** 2026-01-27

**Authors:** Melissa Murillo Timoschenco, Habiba Nur, Martha Archuleta, Mario Suarez, Abiodun T. Atoloye

**Affiliations:** 1 Department of Nutrition, Dietetics, and Food Sciences, Utah State University, Logan, UT USA; 2 School of Teacher Education and Leadership, Utah State University, Logan, UT USA

**Keywords:** Immigrants and refugees, WIC, Utah, Support system

## Abstract

**Background:**

WIC program offers critical nutritional support for low-income families, enrollment and benefit utilization remain suboptimal among eligible immigrant and refugee participants. Understanding the unique challenges they face is essential to improving access, equity, and the effectiveness of WIC services.

**Methods:**

This qualitative study used in-depth interviews to explore the experiences and perceptions of WIC participation among refugee and immigrant families. The study was conducted across communities in Northern Utah and included 25 participants recruited through WIC clinics, community organizations, and social media. Interviews focused on participants’ experiences with WIC enrollment, benefit utilization, and their suggestions for program improvement, including issues related to program awareness, navigation challenges, cultural relevance, and recommendations for enhancing access and support. All interviews were transcribed, translated as needed, and analyzed thematically using NVivo 14.

**Results:**

Three main themes emerged: i) Enrollment and Awareness: participants primarily enrolled due to financial need, often through personal networks. ii) Challenges in Utilization: language barriers, transportation, WIC card difficulties, and limited culturally appropriate food options were common. iii) Suggestions for Improvement: participants recommended enhanced multilingual support, culturally flexible food choices, stronger outreach, transportation, and improved education on benefit use.

**Conclusion and implications:**

Addressing structural and cultural barriers through targeted education, language services, and inclusive support systems and policies can enhance WIC participation and promote equity. Future research should explore the long-term impacts of these efforts and the factors that influence sustained program utilization

## Introduction

Food insecurity is a critical issue in the United States, disproportionately affecting marginalized populations, including immigrants and refugees. The USDA defines food insecurity as a household-level economic and social condition of limited or uncertain access to adequate food [1]. In 2023, 15.5% (approximately 18.0 million) of U.S. households were food insecure, and 17.9% (6.5 million) were families with children [1]. Food insecurity is associated with multiple challenges, including difficulties in accessing nutritious food, limited neighborhood access to affordable fruits and vegetables, and economic constraints that force families to prioritize other financial obligations over food purchases [2, 3].

Immigrants and refugees experience food insecurity at higher rates compared to other populations [4–6]. Structural barriers such as language difficulties, unfamiliarity with the food environment, and economic instability exacerbate their risk [7, 8]. Research has shown that refugee households with young children are particularly vulnerable [8], and undocumented Latino immigrants face additional challenges due to restrictive immigration policies, employment conditions, and limited access to social support systems [9]. These factors collectively contribute to food insecurity and create difficulties in maintaining adequate nutrition for their families.

To address food insecurity among low-income families, the U.S. government has implemented various nutritional assistance programs, including the Special Supplemental Nutrition Program for Women, Infants, and Children (WIC) [10]. WIC is a federal grant program but administered differently across states and local agencies [11]. WIC aims to safeguard the health of low-income pregnant women, infants, and children at nutritional risk. Participants receive benefits to purchase WIC-approved foods designed to supplement essential nutrients [12]. Studies have demonstrated that WIC is cost-effective and beneficial, improving birth outcomes, dietary quality, overall health, and cognitive development [13–18] while reducing racial and ethnic disparities in infant health outcomes [19, 20].

Despite these benefits, WIC enrollment among eligible individuals has declined in recent years [21]. Hispanic/Latino coverage dropped from 72% in 2016 to 64% in 2020, while Black non-Hispanic participation decreased from 53% to 50% [21]. Refugee children are particularly underserved, with enrollment rates significantly lower (only 62%) than the general eligible population (86%) [22]. Barriers to enrollment vary by immigration status. Undocumented immigrants often report fear of deportation and confusion surrounding public charge policies, while both undocumented and refugee participants experience challenges such as language barriers, misinformation about eligibility, and logistical issues like transportation and childcare [23–26]. Even among those enrolled, WIC benefits are often underutilized [27]. Redemption rates are influenced by factors such as vendor selection policies, food availability, and participant awareness of program benefits. Immigrants and refugees may face additional difficulties navigating the food environment and utilizing their benefits effectively due to cultural dietary preferences, literacy levels, and systemic barriers [7, 9, 27].

## Methods

Addressing these gaps is critical to ensuring that WIC remains an inclusive and effective program. Understanding the experiences of immigrant and refugee families participating in WIC can help optimize program delivery, tailor interventions, and promote equitable access to nutritional support. Moreover, incorporating participant suggestions for improvement can provide practical insights that enhance program relevance, responsiveness, and cultural appropriateness. Thus, the purpose of this study was to assesses the experiences of refugee and immigrant families enrolled in Utah WIC, exploring the challenges they face in utilizing the program and their suggestions for improving access and participation, employing a phenomenological approach [28].

### Study design

This study employed a qualitative design using in-depth interviews to explore refugee and immigrant families’ experiences with the WIC program in Utah. Since the goal was to understand the lived experiences of a focal group, we used a phenomenological approach [28]. This approach emphasizes subjectivity, conscious experience, and the meanings individuals assign to events, allowing participants to describe their experiences in their own words and explore their feelings, thoughts, and interpretations. Ultimately, this method focuses on uncovering the essence of lived experiences from the participants’ perspectives [29]. This study was conducted from May 2024 through August 2024.

### Participants and recruitment

A total of 25 refugee and immigrant families participated in the study. These families were drawn from a larger mixed-methods investigation into WIC benefit-use patterns among refugees and immigrants. For the main study, participants were purposively recruited based on their refugee or immigrant status and enrollment in the State of Utah’s WIC program; those who volunteered for the qualitative interviews elected to take part in this in-depth follow-up. This purposive sampling method was consistent with the phenomenological approach [28], ensuring that participants with direct experience of the phenomenon were recruited. Participants for the main study were originally recruited following advice from community leaders from i) local WIC clinics in 4 counties with a high racially and ethnically diverse population in Utah, namely Cache, Davis, Weber, and Salt Lake Counties, using flyers; ii) refugee and immigrant groups, such as CRIC (Cache Refugee and Immigrant Connection), Centro de la Familia, and the Utah Refugee Center; and iii) social media, namely ethnic groups on Facebook, where an e-copy of the flyer was shared.

Ethical approval was obtained from the Utah State University Institutional Revision Board (IRB) under exempt review to ensure the protection of participants’ rights and well-being. Before conducting interviews, informed consent was obtained from all participants through a letter of information outlining the purpose of the study. Each participant was offered a $25 Walmart gift card as an incentive to participate.

### Procedure

The interview questions were developed by: i) conducting a literature review identifying research gaps in WIC benefits use among refugees and immigrants; and ii) gathering feedback on proposed interview questions from three experts on refugee and immigrant and food access research to ensure the questions covers important areas. In line with phenomenology approach, the interviews explored participants’ experiences with the WIC program across four key domains: i) enrollment (e.g., motivation for joining and how they learned about the program); ii) challenges and barriers to accessing and using the benefits (e.g., language, transportation, issues with clinic or store location, and experiences with stigma); iii) food selection and dietary preferences (e.g., specific foods that may not align with cultural practices or preferences, and cultural or dietary adaptation of WIC-approved foods); and iv) suggestions for improvement (e.g., recommendations for enhancing accessibility, communication, and program efficiency). In addition, participants expressed the program’s impact on their health and nutrition. See Table 1 for details of the interview questions.

**Table 1:** Interview Guide exploring immigrant and refugee participants’ experiences with the WIC program

Domain	Interview Questions
Enrollment	1. Can you tell us a bit about your background, including your country of origin and the number of family members in your household? 2. What were the main reasons that led you to enroll in the WIC program? 3. How did you hear about the program? 4. Have you received any assistance or guidance from WIC program staff or community organizations in navigating the program? 5. How effective do you think WIC’s outreach efforts are in reaching immigrant and refugee communities?
Challenges and Barriers	6. What challenges have you encountered while trying to access and utilize WIC program benefits? How did you address them? a. Has language barrier ever been a challenge in understanding or accessing WIC benefits? In what ways? b. Are there any transportation or location-related barriers that have made it difficult for you to access WIC services or redemption locations? 7. Can you share any difficulties you have faced in finding and purchasing WIC-approved foods? 8. Have you experienced any stigma or discrimination related to using WIC benefits?
Food Selection and Dietary Preferences	9. How do you feel about the selections of WIC-approved foods? Are there any limitations you’ve encountered? a. Are there cultural or dietary preferences that you have, which you feel are not adequately met by the program?
Suggestions for Improvement	10. Do you feel that the education or orientation you received adequately prepared you for using your WIC benefits? Do you think additional information or support is needed? 11. What suggestions do you have for improving the education and orientation process for WIC participants in using their benefits at stores? 12. What suggestions do you have for making WIC program information and resources more accessible to immigrant and refugee participants, especially those with limited English proficiency? 13. How can the program better accommodate the diverse dietary needs and preferences of immigrant and refugee families? 14. In what ways do you think the WIC program could be improved by the overall experience of participants in terms of convenience and efficiency? 15. Are there any specific services or support you believe the WIC program could improve to better assist immigrant and refugee families?
Overall Impact	16. How has the WIC program benefitted your family and children’s health and nutrition? 17. If there was one thing you’d like policymakers to know about the experiences of immigrant and refugee WIC participants, what would it be?

The bi-lingual researcher on the team who was responsible for conducting and coordinating the interviews had prior training in semi-structured interviewing using standardized qualitative research procedures [30]. Before commencing the interviews, the researcher practiced conducting an interview with one mock participant. To ensure language accessibility and cultural sensitivity, the bilingual researcher conducted the interviewers with Spanish- and English-speaking participants. For participants who spoke other languages, community health workers served as translators, facilitating communication by translating questions and participant responses back into English. Interviews were conducted until saturation was reached, meaning that no new data were being introduced. All interviews were audio-recorded with participants’ consent to ensure accurate data collection.

### Data analysis

The recorded interviews were transcribed and thematically analyzed using NVivo 14 software. Interviews conducted in Spanish were first translated verbatim by the bi-lingual researcher before analysis. As the study aimed to identify lived experience of the study participants, the qualitative interviews were coded through a culturally sensitive lens. An initial subset of five transcripts were reviewed line-by-line by one researcher, MM who identified significant statements to develop a coding framework. The framework was then reviewed and refined by author ATA, and together they identified meaningful units, which they clustered into themes corresponding to the interview focal areas: experiences, challenges and barriers, and suggestions for improvement. Once consensus was reached on the main themes, the same framework was applied to the remaining twenty transcripts. After all interviews were coded, the associated quotes were reviewed by authors HN and MA to confirm alignment. Any necessary adjustments were then incorporated based on their feedback before finalizing the thematic analysis.

## Results

### Demographics

Participants (*N* = 25) were all female and represented diverse countries of origin, including Somalia, Congo, Eritrea, Spanish-speaking communities (Mexico, Guatemala, and Peru), Nigeria, and Ghana (Table 2). The majority were between 25 and 34 years old (64%), married or living with a partner (68%), and had been enrolled in WIC for more than two years (60%). On average, households included 5.5 individuals with 2.4 children under five years of age. They have been living in the country for six months to nine years.

**Table 2:** Characteristics of participants (*N* = 25)

Characteristics		n (%) or Mean ± SD
Country of origin	Somali	6 (24)
	Congo	6 (24)
	Eritrea	6 (24)
	Spanish-speaking (Mexico, Guatemala, Peru)	5 (20)
	Nigeria	1 (4)
	Ghana	1 (4)
Age group	18–24 years old	4 (16)
	25–34 years old	16 (64)
	35–49 years old	5 (20)
Marital status	Married/living with partner	17 (68)
	Separated/divorced/single parent	8 (32)
Education	No formal education	4 (16)
	< 8^th^ grade	3 (12)
	9^th^ − 11^th^ grade	7 (28)
	High school degree	8 (32)
	Bachelor’s degree	2 (8)
	Graduate degree	1 (4)
Annual household income	<$25,000	14 (56)
	$25,000-$34,999	5 (20)
	$35,000-$49,999	6 (24)
Employment status	Not working/in school	17 (68)
	Part-time	3 (12)
	Full-time	5 (20)
Time in WIC	< 1 year	6 (24)
	1–2 years	4 (16)
	> 2 years	15 (60)
Household size		5.5 ± 2.6
Children < 5 years		2.4 ± 1.4

The main themes that emerged from the data included *factors related to WIC enrollment*, *challenges utilizing the benefits,* and *suggestions for improving the program experience*.

### Themes related to WIC enrollment

#### Reasons for joining WIC

When asked the reasons for enrolling in the WIC program, most of the participants mentioned the need for additional support in managing household expenses. Many described WIC as a crucial resource that helped alleviate financial pressures related to providing essential nutrition for their families. For several participants, WIC served as a necessary form of assistance, particularly when it came to covering basic grocery expenses. One participant mentioned how WIC made it possible to buy formula, commenting:

*The main reason is the need, especially as a mother. We can’t work right away because we have to take care of our babies. That’s why I say this program has been incredibly beneficial. Honestly, since I’ve been part of it, there have been very few times I’ve had to buy a can of formula with my own money. When my daughter was little—she’s now growing up, like Lucita—I breastfed her and also supplemented with formula.*

The program also helped participants meet other financial needs, such as paying bills, allowing them to reallocate their limited income.

Many said that their salaries were insufficient to cover both food and household costs. Even though they have a job, they still faced difficulties in making ends meet. One participant expressed frustration, saying:

*Because the amount I’m getting as a graduate research assistant is not enough to buy food.*

In some cases, unemployment led individuals to seek out WIC. The lack of income made the need for immediate support urgent, turning WIC into a crucial resource during a challenging time.

*“The main reason was because I lost my job, they made me leave the company where I worked. Well, once again … I needed the service”*, one participant commented. 

Additionally, several participants reported that they were referred to WIC by healthcare professionals or social service providers. These suggestions often came from trusted sources, including hospitals, doctors, or caseworkers, which encouraged participants to pursue enrollment with confidence. For example, one person shared:

*When I had my first daughter, then I got enrolled into the WIC, and then when I got pregnant with my second one they [referring to hospital staff] also enrolled me in the program because they told me it would be really hard to buy them food.*

#### WIC awareness

Participants reported that they learned about the program through several avenues, primarily from personal connections or community networks. Additionally, many refugees first became aware of the program through resettlement agencies and case workers, who are responsible for connecting newly resettled individuals to available resources such as WIC and SNAP benefits.

The majority of interviewees (*n* = 13) heard about WIC from someone within their social circle. Friends and family members played a significant role in spreading awareness of the program. In particular, one participant noted:

*About a month into my pregnancy, a friend told me about a place where they check things like iron levels for pregnant women and provide nutritious food, including protein. So, I decided to go, and they accepted me right away.*

In addition, the community support circle also helped raise awareness among respondents. Professionals such as case workers, doctors, and hospital staff were important sources of information. One participant said:

*My caseworker helped me sign up. I didn’t know about WIC until she mentioned it.*

One family also became aware of WIC through flyers received by mail. 

#### WIC efforts to reach out to immigrants and refugees

The perceptions of WIC’s outreach efforts particularly within immigrant and refugee communities varied among respondents. Some participants believed that WIC’s outreach was sufficient, feeling well-informed and adequately supported by the program.

However, several participants felt that WIC’s direct outreach to immigrants and refugees was lacking. As mentioned, most have learned about the program through external networks, such as friends, family or other professionals rather than WIC itself. As one respondent shared:

*“You get the information from the community, so not WIC.” Another participant said: “Through the case manager. It’s the caseworker who makes the connection between the refugees and the WIC.”* Other mentioned: *“There’re many people don’t know this program.”*

Many acknowledged that personal connections played a crucial role in learning about the program. Social networks and community organizations, such as the Somali community or refugee offices, were the main source of information when spreading the word.

Participants also appreciated the personal and individualized treatment they received from WIC staff: *“I think they’re really good at it. When I had my baby girl, I received a text message right after—it felt very personal. They texted me about my due date, and a day or two after I gave birth, they sent a message saying ‘congratulations’ and asked if I needed help with breastfeeding. I really did, because I was struggling. So, I replied, explaining the issues I was having. They offered to set up a meeting, and I went to the office where they showed me how to hold her and get her to latch properly. It was really helpful. So yes, I think they do a great job with that.”*

Even though it was reported that the outreach effort is lacking, the program was seen as a valuable resource for immigrant and refugee families, with participants describing the program as “very helpful” in meeting their needs considering their vulnerable situation. One participant expressed gratitude, saying, *“I think it’s good because they did help us. Personally, they helped us right from the start. I thought they might not help because we had just arrived, or we might not qualify. But we showed our ID and other documents, and they told us that we qualified. They even gave us the card on the same day*.” And another said: “*it is very good because it supports people who sometimes have no chance, not even a job. And yes, well, I think it is a good program.”*

### Themes related to challenges while utilizing the program

#### Challenges while utilizing the program

Participants highlighted various challenges and barriers they faced while navigating the WIC program, where the most common barriers reported were language issues, transportation, and difficulties with the redemption system.

Firstly, language posed a significant challenge for many participants considering that English is not the first language for any of the participants. Understanding program instructions, reading materials, and communicating with the WIC staff or store personnel were often difficult. One participant expressed frustration, saying:

*Because of not being able to read and speak English, especially in the past, it was, it was really hard to even find the grocery allowed to buy, now it’s getting a little better.*

*“Language has been a problem because when I buy, they talk to me, they told me something and I saw them take the food back. I don’t understand what they’re saying. The communication is hard, I don’t understand what they’re saying,*” another participant stated.

A participant also expressed: “*Well, for me I think the biggest challenge here is the English issue. Yes, sometimes I’ve had to deal with the fact that there isn’t a pediatrician or nutritionist who doesn’t speak Spanish, but since I’m almost always with my daughter, she helped me with that.”*

Secondly, access to transportation was also mentioned by several participants. While some found the WIC location convenient and easily accessible, others struggled with getting to and from appointments or stores, especially having to take the children or carrying the bags from the store.

A participant said: “*Specially in the winter and with the snow it is very hard to even use the bus with the kids to go to the clinic.*” Another one mentioned: *“That’s a problem. It’s difficult. I’m walking to Smith and walking back with all the bags.”*

Another participant explained: “*It was the first time, and I remember I had to take my daughter. I had no choice but to take two buses, and I made it there fine, even though I was still living in the south at the time. That day I arrived late, but the pediatrician was very understanding. She asked why I was late, so I explained that I was coming from North Logan. I thought, ‘Oh no, they’re not going to see me now,’ but she made an exception and did see me after all.”*

Finally, navigating the WIC card system presented several difficulties for participants, particularly when it came to PIN numbers, finding approved products, understanding the redemption process, and the introduction of the new card. One major issue cited by many participants was confusion when trying to locate WIC-approved products in stores. Discrepancies between the items listed in the app and what cashiers would approve created further confusion, with one participant sharing*: “The app shows one thing, but when I get to the cashier, they say it’s not covered.”* Some participants also reported issues with understanding how to redeem their benefits, while others struggled to remember their PIN number or felt the process was overwhelming. One participant commented:

*As of today, I don’t have any challenges, but when we first started, it was difficult because we were new to it. Finding the WIC-approved items was tricky, especially at Walmart, since there are so many products and it was hard to know where to look. The first few times we shopped, it was confusing because we only use WIC once a month, so it took some time to figure out. Now that we’ve been using it for a while, we know exactly where to go, and it’s much easier. Winco, for example, has WIC-approved signs or posters, which make it clearer to find the items. That helps a lot.*

#### Problem-solving and overcoming challenges

Despite these challenges, participants were able to find ways to better navigate the program more effectively by seeking help or using available tools. Several participants acknowledged that the WIC app was helpful in overcoming some of the program’s challenges, such as tracking benefits and identifying approved products in stores. Additionally, participants frequently relied on external sources of support to overcome program-related challenges. The Somali community, caseworkers, store staff, and WIC staff were mentioned as the major key sources of assistance.

A few participants demonstrated resourcefulness in addressing their difficulties, choosing to rely on themselves when problems arose. One participant noted, *“I figured it out on my own after some trial and error.”*

#### Selection of WIC-approved foods

Participants shared mixed feelings when asked about the selection of WIC-approved foods, expressing both satisfaction and frustration with the program’s offerings.

On one hand, some participants found the approved food options adequate for their needs, with one noting, *“The food we get is good enough, it helps a lot.”* Respondents agreed that the products are healthy, nutritious and offer a variety of vitamins and minerals.

On the other hand, they expressed dissatisfaction with the limited flexibility of the WIC-approved food options. For some, the ‘fixed menu’ system left them with unused products that could not be exchanged for more practical alternatives. As one participant explained:

*Currently, it seems like there is a fixed menu or set of products available for everyone. But, considering that people come from diverse backgrounds, allowing for some flexibility in the categories could make the program better fit individual needs and preferences.*

Others mentioned the limited variety of foods covered by WIC, citing specific items that were unavailable. Many participants listed some key products they would like to see included, such as preferred types of cheese and milk, flour, bread, sugar, spices, meat, chicken, fish, and palm oil. Participants also reported that the amount of certain staple items that are not sufficient for the family, such as milk, vegetables, fruits, eggs, and grains. One participant shared, *“The milk and eggs aren’t enough for the whole family, and we have to buy more.”*

Participants also mentioned that the items selected are healthy but not of their cultural preference. While participants recognized the nutritional value of the items provided, some felt that the offerings were not inclusive of culturally preferred food. As one participant explained, *“It’s really good because it meets all the nutritional and dietary needs, but it doesn’t take individual preferences into account. It doesn’t consider what people like.”*

### Themes related to suggestions for improving the program experience

Participants offered a wide variety of suggestions to enhance the WIC program’s effectiveness, focusing on areas such as education and orientation process, accessibility of resources and information, convenience, and dietary inclusivity.

#### Education and orientation process

Participants highlighted the need for additional educational and orientation efforts to help beneficiaries better understand how to use WIC benefits. Several respondents suggested providing information in multiple languages, emphasizing the importance of translation services.

In-person education sessions were also mentioned as a helpful resource to improve the orientation process, along with online resources for those who prefer remote access. Some participants recommended employing more diverse staff members to reflect the program’s multicultural population. As one participant noted, “It would help if there were someone who spoke our language at the store.”

Additionally, respondents suggested more education around specific topics, such as how to use the app, understanding portion sizes, and feeding children. One participant expressed the need for more clarity on product sizes, stating, *“Explaining how to redeem the sizes correctly would be helpful.”*

#### Make resources and information more accessible

Participants suggested that WIC increase its outreach and make information more accessible, particularly through the use of social media and community-based efforts. One participant remarked:

*Social media is widely used, with many people on Facebook, so it would be beneficial to use it for outreach. Additionally, text messaging could be effective, as I often receive texts from companies whose sources for my number are unclear. Facebook groups in Cache Valley, where people frequently give away free items, could also be a great way for advertising. Programs like the Food Pantry and the Little Lamb diaper program already use these methods, so leveraging them could be advantageous for a large program like this.*

The need for more widespread translation services was mentioned by almost every respondent, emphasizing the importance of communicating in a way that beneficiaries can easily understand.

*“Interpreters are very important. I agree with the need for interpreters because many people can speak but may not be able to read or write effectively. For example, I might be able to write correctly sometimes, but I might misspell words or find reading difficult. Having an interpreter can be very helpful in these situations,”* one participant said.

Another one mentioned: *“There are videos and booklets in Spanish, but I think it would help if there were more Spanish-speaking staff. Right now, only two people speak Spanish fluently, and the others try, but it can be difficult.”*

#### Convenience

In terms of making the program more convenient and effective, several participants provided suggestions to improve their experience, such as extended hours for WIC offices, with one participant explaining: *“The hours could be improved because it’s usually like 8 to 6, but for working families or for families with kids who are at school, they have to go only before the kids go to school or after they get back home, which is in the late evening. So not enough hours, maybe expanding.”*

Another suggestion mentioned by several respondents regarding convenience was offering transportation services, as many participants struggled with access to reliable transportation. One participant proposed:

*Having transportation available would be good. Some people can’t go to WIC appointments because they don’t have transportation. And WIC usually requires that you come with your child, which makes it even harder for those without transportation to attend.*

While some participants found the program already efficient, others suggested phone-based assessments as a way to reduce in-person visits.

#### Target diverse dietary needs

Some participants expressed a desire for the program to better target the diverse dietary preferences of WIC beneficiaries. Several recommended conducting surveys to gather feedback from WIC participants on their needs and preferences, particularly for children’s food choices. One participant suggested:

*To accommodate more to their preferences, to the kids taste because it depends on the kid taste. If the kid does not like the food, then you could tell the WCI staff to change it.*

Expanding the number of stores, such as ethnic foods, was another common suggestion. Participants also wanted the ability to trade the amount of certain items they received, especially when they couldn’t fully use the products. *“If we could trade some of the items for what we actually use, it would be more helpful,”* one participant explained.

#### Messages for policymakers

Participants were asked if they had something to say to policymakers about refugee and immigrant participants, what would that be? Several participants wanted to thank policymakers for the assistance WIC provides, expressing appreciation for the program’s positive impact on their families. One participant said:

*Thank you. Thank you very much, because they support us a lot, some food we have no the money to buy it, but then they provide for our kids. And our kids grow up strong and healthy. So that I’m very, very thankful for them.*

Other expressed: *“I’d say they’re doing a great job. They keep improving the program, making things easier with the card and the app, and they don’t discriminate against anyone. They’ve helped us as immigrants, and I’m grateful for that.”*

Furthermore, participants suggested being more responsive to the needs of diverse populations. For example, greater cultural inclusivity and the provision of translators to improve communication with non-English-speaking beneficiaries.

Participants also mentioned continuing and even expanding the WIC program to cover more demographics, such as seniors or older children. Some participants also recommended increasing advertisement and outreach efforts to ensure that more families are aware of WIC. *“They should also increase public awareness, especially for those who may not have access to social media. For instance, they could distribute flyers at places like Walmart or use billboards to reach more people.”*

## Discussion

The objective of this study was to understand refugees and immigrants’ experiences in WIC, including challenges and barriers utilizing the program, and their suggestions on how to improve their utilization of the program benefits. The main themes that emerged from the data included: factors related to WIC enrollment, challenges utilizing the benefits and suggestions for improving the program experience.

This study found that for these groups, WIC enrollment is primarily facilitated by referrals by health workers or social service providers, though others include hospital staff, doctors, or caseworkers. Unlike native-born populations, the reliance on referrals is particularly significant for refugees and immigrants to navigate complex healthcare and social service systems in a new country [31]. Furthermore, it is not surprising that the majority enroll in WIC for financial support, as the program is designed to assist low-income families by providing essential nutrition assistance. They rely on WIC to access formula and basic groceries, alleviating financial strain while ensuring proper nutrition for their children. This finding is consistent with a previous multistate study on WIC participants (*n* = 31,225 in English and 7,396 in Spanish), which examined post-pandemic food security, reasons for participation, experiences with services and shopping for WIC foods, and perceptions of the cash value benefits (CVB), EBT card, and app. Participants reported enrolling in WIC for the CVB and other WIC foods [32].

Regarding WIC awareness, participants reported learning about the program through personal connections, community networks, caseworkers, and healthcare providers. While these channels serve as key outreach efforts to immigrants and refugees, perception of these outreach efforts varied; some felt WIC’s outreach was sufficient, others believed direct outreach were lacking. A mixed-method study evaluating military families applying for childcare financial assistance found that misinformation and lack of program awareness created barriers to WIC participation [33]. Consistent with the present study, that research also noted that word-of-mouth was the primary source of information, often spreading misconceptions about income eligibility and age cut-offs [33]. Collectively, these findings highlight the critical need for broader and more systematic outreach efforts beyond reliance on social networks, specific employment groups, language communities, or age groups to improve WIC enrollment among eligible families.

In terms of themes related to challenges while utilizing the program, language difficulties, transportation issues, and confusion navigating the WIC card system were challenges reported. These challenges align with previous research on general WIC participants. For example, a study in Massachusetts exploring reasons for incomplete benefit redemption and early dropout found that EBT card issues and barriers to clinic access were significant obstacles for WIC caregivers, though the study did not focus on immigrant participants [34]. Similarly, research on WIC participants in California reported that nine in ten experienced difficulties shopping for WIC foods, particularly in locating eligible items, which aligns with the card system confusion reported in the present study [32]. Another qualitative study across Texas, North Carolina, Oregon, and Illinois found that general WIC participants faced challenges in identifying WIC-allowable items and experienced perceived stigmatization during checkout [35]. A cross-sectional study in Missouri (*n* = 2,244) found that almost 40% of WIC participants faced barriers related to technology access, transportation, clinic hours, and wait times, while 35% cited challenges with clinic location, and nearly 30% reported childcare-related difficulties [36]. While these barriers are common across WIC beneficiaries, the present study highlights additional challenges unique to immigrant participants, such as language barriers and difficulties understanding program materials, which may further complicate their WIC experience. Along these lines, a recent survey among refugee and immigrant participants highlighted additional barriers to utilizing benefits, including difficulties understanding the redemption system, language barriers, inconsistent stocking of WIC-approved products, unfamiliarity with certain foods, discrepancies between what the app and cashiers approved at checkout, limited brand options, and inefficiency of some store staff in assisting with WIC purchases [37]. This might indicate a need for clearer communication and orientation to improve support systems for participants and thereby minimize or avoid the above-mentioned issues that lead to incomplete benefit utilization to improve benefit utilization and retention among immigrant families.

Problem-solving and overcoming challenges involved the use of WIC app, seeking help from community support networks, and demonstrating resourcefulness. A 2022 study reported that while technological tools like EBT cards and smartphone apps improved shopping experience by reducing stigma and simplifying eligibility checks, challenges remained [34]. Mislabeling of WIC-eligible items and poor in-store cell service caused confusion and embarrassment at checkout [34]. This suggests that despite technological advancements, participants still face difficulties due to technical issues, which can disrupt the navigation of the WIC program. This highlights the need for improved store staff training and enhanced app functionality to better support participants, ensuring they can navigate these challenges independently.

Participants expressed satisfaction with the WIC-approved products offered but noted limited flexibility and lack of culturally preferred foods. While they appreciated the healthfulness of the products provided, some did not align with their cultural preference. These findings align with a statewide WIC survey among 3,167 parents/caretakers, which found that good nutrition and formula were the most valued benefits. However, Hispanic respondents reported that certain WIC foods (e.g., dry beans, milk) did not align with their dietary preferences, highlighting the need for greater cultural adaptability in food packages [38]. Furthermore, participants expressed insufficient amounts of products and limited variety. A common misconception among participants was that WIC benefits were intended to provide for the entire family, rather than being specifically designed to meet the nutritional needs of the eligible individual (e.g., the child or pregnant/postpartum mother). This misunderstanding may contribute to the perception that the provided amount is insufficient. Addressing this issue through improved participant education, such as clearer explanations during WIC appointments, flyers, or culturally tailored messaging, could help families better understand the program’s purpose and plan their household food purchases accordingly.

Another concern was that the ‘fixed menu’ system resulted in unused products that could not be swapped for more practical or preferred options. These challenges are consistent with those reported in previous research, where participants expressed low satisfaction due to insufficient funds, especially for fruits and vegetables, food benefit inflexibility, as well as in-store item mislabeling [32, 34, 35, 37].

Lastly, participants’ suggestions for improving the program experience focused on education and orientation processes, including multilingual educational resources, in-person sessions, and diverse staff to cater to the multicultural population. These suggestions were closely related to challenges with understanding the card system and app, as well as the duration participating in the program. Participants, especially newly resettled refugee or immigrant, might require more time, orientation and guidance to become comfortable with the program and learn to navigate the redemption system. These findings together point to the need to enhance targeted education and support for new enrollees to best utilize their benefits and optimize their experience with the program. In addition, to make resources and information more accessible, participants recommended using social media and text messaging for outreach and emphasized the importance of more translation services and accessible communication methods.

In terms of convenience, some expressed the need for extended WIC office hours and provision of transportation services to make the program more accessible for families with limited transportation options. Similarly, a large 2019 study by Frank et al. (*n* = 2,244) found that nearly 40% of participants had difficulties taking time off work, and 35% faced barriers related to clinic location, highlighting the need for more flexible service options [36]. Additionally, respondents in another large, multi-stage study suggested app features for appointment scheduling and benefit expiration reminders, as well as the ability to use self-checkout aisles to streamline their shopping experience [32].

To better target diverse dietary needs, participants emphasized the need for more culturally inclusive food options, greater flexibility in food selection, and expanding the number of stores that accept WIC. These findings align with a study in which participants also recommended expanding shopping options, particularly at farmers’ markets [32].

This study confirms several barriers commonly reported in the broader WIC population, such as difficulty identifying eligible products [32, 35, 39], stigmatization [32, 35, 39], insufficient amount of some products [39], limited transportation [34, 36], and confusion about the EBT card system [34, 36]. It also highlights unique challenges specific to immigrant and refugee populations such as language barriers and cultural complexities. Additionally, it underscores the importance of external networks; not only to raise awareness about the program but also to provide initial support in navigating the complexities of social service systems for a smoother experience. By focusing on these underrepresented groups, this study adds important insight not often captured in broader WIC studies. Participants’ suggestions for multilingual education, culturally sensitive food options, and expanded outreach strategies provide meaningful feedback that could inform more inclusive program development. Notable, this study also highlights direct feedback for policymakers, which underscores the necessity of policies that enhance WIC flexibility, access, and cultural responsiveness. These results point out the ways that strengthening WIC benefits not only supports participating families but also contributes to broader public health goals, including reducing healthcare costs and improving child health outcomes nationwide.

This study has some limitations that should be considered when interpreting the findings. First, the sample size was relatively small and recruited from a specific geographic region, which may limit the extent to which results are transferable to other settings. Participants’ experiences may not reflect those of families in other areas with different local WIC policies or support systems, particularly since the program is a federally funded but administered with some variation at the state and local levels. Second, language barriers and the use of interpreters in some interviews could have influenced participants’ responses or led to nuances being lost in translation. Finally, while this research explored the experiences of WIC participants, it did not include the perspectives of WIC staff or administrators, which could have presented a more rounded view of the problems and potential solutions to increasing benefit use. Nevertheless, this study provides valuable information about the experiences of refugee and immigrant families in WIC, a group often underrepresented in the literature. The qualitative approach allowed for rich, detailed descriptions of participants’ challenges and suggestions, yielding specific recommendations for increasing program accessibility and cultural responsiveness.

## Implications for reasearch, practice and policy

Participants expressed satisfaction with the nutritional value of WIC-approved foods but emphasized dissatisfaction with the program’s inflexibility, mentioning cultural misalignment and lack of appropriate personalization as major barriers. These claims are supported by previous studies underlining the role of a food package with more cultural emphasis and expanded program flexibility.

This study highlights the critical role of education, orientation, guidance, and support systems in maximizing WIC benefit utilization among participants from refugee and immigrant communities. Enhancing orientation programs, expanding language support, and tailoring food preferences to diverse cultural needs can improve the accessibility of WIC services for these populations, and the allocation of resources more effectively. Moreover, policy considerations should focus on addressing structural and technical barriers such as transportation and technology access to ensure equitable program participation.

In conclusion, while the WIC program is successful in safeguarding the health of low-income women, infant, and children at nutritional risk by providing essential nutritional support, this study highlights the need for strategic modifications to address the unique needs of diverse populations. Future research should explore longitudinal impacts of program enhancements and investigate additional demographic and psychosocial factors influencing WIC utilization. This effort is crucial to promote inclusive practices and minimize health disparities

## Data Availability

The datasets generated or analysed during the current study will be made available upon request.
